# Biomaterials in urinary incontinence and treatment of their complications

**DOI:** 10.4103/0970-1591.65394

**Published:** 2010

**Authors:** Philippa Sangster, Roland Morley

**Affiliations:** Department of Urology, Kingston Hospital, Galsworthy Rd, London KT2 UK

**Keywords:** Biomaterials, female, incontinence, injectables, slings

## Abstract

Biomaterials integrate with the anatomy and provide support to the weakened area. They are generally synthetic, but natural substances are also used. These substances are being increasingly used in stress urinary incontinence. This article discusses the various biomaterials, minimally invasive techniques, and recent advances for the treatment of female stress urinary incontinence. In addition, their complications and subsequent management are explored.

## INTRODUCTION

A biomaterial is any material, natural or man-made, that comprises whole or part of a living structure or biomedical device which performs, augments, or replaces a natural function. Female stress urinary incontinence (SUI) is a significant health problem with considerable social and economic impact. Biomaterials, primarily synthetic, are often utilized to augment surgical correction. Repair with biomaterials included peri-urethral injectables, intra-vesical treatments and midurethral support to function against weakened connective tissue caused by injury, abnormal collagen metabolism, or genetic predisposition. Even though efficacy rates are high, the potential for complications, such as erosion, are great without comprehension of the inherent characteristics of each graft material.

In regards to sling materials, low-weight, macroporous, monofilament synthetic grafts and non-cross-linked biologic grafts are examples of biomaterials that implant reasonably well with host tissue. The theory of using mesh or grafts to improve the structural integrity in surgical anatomy is not novel. Prosthetic devices for abdominal hernia repairs have been found as early as ancient Egypt. What is novel is the rapid expansion and marketing of new synthetic and biologic mesh materials in modern medicine.

The concept of slings for urethral support was first introduced in 1907 by Von Giordano. McGuire and Lytton reintroduced the procedure to urologists in 1978 using the combined abdominal and vaginal approach incorporating rectus fascia.[1]

In the past decade, sling surgery has become the preferred technique for the management of female stress urinary incontinence. A greater understanding of the pathogenesis of stress urinary incontinence and a greater durability and effectiveness for sling surgery has allowed this technique to become the benchmark for treatment of female stress urinary incontinence. As a consequence, a multitude of products have been developed using various techniques and materials to perform sling surgery. To minimize the morbidity of graft harvest, biologic and synthetic graft materials (biomaterials) have been increasingly used in sling surgery. Decreased perioperative pain and hospital stay have been associated with the use of graft substitutes.[2]

## THE IDEAL MATERIAL

The ideal biomaterial would be chemically and physically inert, sterile, noncarcinogenic, mechanically strong, not physically modified by the body tissue, readily available, inexpensive, and have minimal risk of infection and rejection. In incontinence surgery, once healed the graft should restore normal anatomy and function to the pelvis, and be equally durable to autologous tissue. In addition the material should remain long enough for incorporation of the surrounding host tissue. It should withstand mechanical stress and shrinkage, be pliable and easily manipulated during surgery, causing minimal surrounding reaction.[3] Although not completely ideal, many of the available biomaterials have certain characteristics that fulfill such requirements.

## NATURAL BIOMATERIALS

### Slings

Biomaterials include autologous grafts (tissue harvested from the patient), allografts (tissue obtained from a source other than the recipient but from the same species), and xenografts (tissue obtained from a species different from the recipient).

### Autologous

The commonly used materials here are rectus fascia and tensor fascia lata. Rectus fascia is easily harvested, even in patients with multiple abdominal operations. It has been shown to be durable and rarely causes urethral erosion. FitzGerald *et al*. reported the histological changes after sling placement and found extensive remodeling with increased fibroblasts and connective tissue on biopsy specimens.[4] Initial data comparing the primary outcome of continence with rectus fascia versus TVT showed both to be equally effective. In addition, symptom scores related to incontinence surgery as well as simultaneous correction of cystocele were comparable in the two groups.[5] The success rates of autologous grafts at 33-52 months have been quoted at 70-91%.[6,7]

The disadvantages are the longer operative time, higher surgical morbidity, postoperative pain and longer recovery. In addition rectus fascia may be scarred and thickened owing to prior operations.

In regards to the fascia lata, this tissue is an easily obtainable long graft that is generally unscarred and of uniform thickness. The length means it is easier to achieve adequate tension on the sling. As there is no abdominal incision, recovery time is less, and there is no risk for abdominal hernias. However, it is a longer operative time in an area unfamiliar to the urologists.

### Allograft

Allograft tissue such as cadaveric human fascia lata, dura or even dermis has been used. As discussed the use of nonautologous materials is popular and attractive because it decreases operative time and avoids the possible morbidity associated with a second surgical site. These tissues do carry the risk of infectious disease transmission, in particular Creutzfeldt-Jakob disease and other prion transmission-related illnesses.[8] There have been conflicting reports in the literature on whether outcomes are compromised with the use of cadaveric fascia.[9] Although prions are resistant to treatments such as radiation that target nucleic acids, denaturing agents may destroy them. Solvent dehydration of the graft removes the prions without compromising tissue integrity. It has been reported that grafts undergoing a freeze-dried technique may have diminished tensile strength and tissue consistency when judged against solvent-dehydrated fascia and autologous fascia.[10]

Simsimans *et al*.’s study[11] assessing the outcome of suburethral slings by type of sling material over a six-year period found that patients undergoing surgery with either allograft or xenograft materials were more likely to experience recurrence of their incontinence than women undergoing the same procedure with autograft materials. The majority of other studies only provide short-term data (less than two years), and quote success rates from 65–98%.[12] Several groups have found intermediate-term failures six months postoperatively, the reason of which is unclear. Possible explanations include a lack of standardization in the technique of tissue processing and surgical preparation, as well as differences in allograft tissue-remodeling by the patient.[13]

### Xenograft

Porcine dermis has been used in incontinence surgery, however, there are no long-term randomized controlled studies investigating their use. This acellualr graft is made up of collagen and elastin fibers which provide a matrix for which new tissue and cells can be supported on. Although it is assumed that there should be no immune response to this structure, however, a small comparative study investigating the body’s histological response to a variety of materials, showed that porcine grafts were most likely to be encapsulated within the host.[14] Medium-term data have reported success rates of 94 %[15] on Grade 3 and 4 cystoceles, with minimal complications.

A three-year follow-up comparing porcine dermis and TVTs, complications and patient satisfaction, found a cure rate of 82.4% vs. 88.3%. There was no significant difference in the complication rates and satisfaction levels between the two groups.[16]

Processed porcine small intestine submucosa (SIS) was approved for deep human implantation over 10 years ago. Early studies showed that SIS graft material caused local host tissue cells to infiltrate its substance and essentially replace it within 90 to 120 days. The manufacturing process transformed the intestinal submucosa into an acellular collagen matrix. This implanted graft was then remodeled and changed into host tissue. Both the SIS graft and the subsequent host tissue were biocompatible and resistant to infection as well as being strong and durable. These properties indicated that SIS could be an excellent material for use in sling surgery.[17,18]

Early studies of 152 patients who underwent sling surgery using an SIS sling anchored to the pubis with bone screws, showed that 142 women (93.4%) were relieved of their SUI after a median follow-up of 2.3 years.[19] There were no cases of sling infection, erosion, or rejection during the four-year follow-up of this series. However, all patients who were taking anticholinergics preoperatively continued to do so after surgery.

Subsequent data where SIS slings were inserted with a curved ligature carrier to create a tract between bilateral suprapubic stab incisions and a 2-cm mid-urethral vaginal incision has shown less successful statistics.[20] Only 27 of the 34 women (79%) at the two-year follow-up reported that their stress incontinence was cured. Although three (9%) of those with no complete resolution were pleased with their results, as there was enough of an improvement to allow them to wear an average one or fewer pads per day. One patient developed *de novo* urge incontinence. Unlike the first study three patients (9%) developed suprapubic inflammation at 10, 21 and 45 days after surgery; all resolved, but one had a recurrence of SUI. No other complications were recorded.

### Synthetic biomaterials

There are a variety of synthetic materials, which come in many different forms with distinct characteristics intended for specific functions. Notable advantages of a synthetic mesh include the lack of potential infectious disease transmission and high tensile strength. In addition these meshes are readily available, cost-effective, and do not require harvesting, therefore reduce operative risks.

Synthetic materials can be absorbable or non-absorbable. Absorbability has the benefit of encouraging postoperative fibroblast activity, as well reducing infection rates and causing minimal harmful effects to surrounding tissue. Once inserted, macrophage activation leads to mesh absorption and later recycling of byproducts into new collagen fibers.

Synthetic materials can be further described as macroporous or microporous. Pore size greater than 75 mm is regarded as macroporous, those less than 10 mm are thought of as microporous. The pore size controls which cells (macrophages versus bacteria) can enter the graft material. In theory, the level of resistance to infection when using multifilament fibers is proportional to the small interstitial spaces between fibers. Closely woven mesh or multifilament fibers may offer a safe refuge for small bacteria and could exclude macrophages and leukocytes. In contrast, loosely woven fibers can increase the levels of ingrowth and neovascularization. A tightly woven and large-diameter filament mesh reduces pliability, which may contribute to migration, extrusion, or erosion.[3]

Another point to consider is that if the sling becomes integrated with the surrounding tissue, although this should strengthen the support, a solid scar formation may cause problems if the need to remove the sling arises. Finally, meshes may also contain additives and coatings that may impact acceptance by human tissue.

### Synthetic slings

Synthetic mesh materials have been classified on the basis of pore size and the filamentous nature of the material. Type I meshes are macroporous and monofilament.

Type II meshes are microporous with pore sizes less than 10 mm. Type III meshes are macroporous meshes with multifilamentous components (containing pore sizes less than 10 mm). Type IV meshes are ‘coated’ biomaterials that have submicronic (less than 1 mm) pore size.

### Polyethylene tetraphthalate mesh

Polyethylene was first synthesized in 1898 by von Pechmann; the industrially practical first form of this material was discovered in 1933 by Gipson and Fawcett. This polymer consists of long chains of the monomer ethylene, and is classified into several categories based on density and branching. It is used in the multifilament, Type III mesh also known as Mersilene^®^ (Ethicon, Somerville, New Jersey, USA) was used more commonly used in the last decade. The woven design of this mesh meant it could be trimmed without unraveling or losing its bidirectional elastic properties.[21] The use of Mersilene^®^ mesh and the gauzehammock technique was further popularized by David Nichols in 1973[22] as the definitive treatment of severe recurrent SUI. Initial short-term data showed encouraging results with objective cure rates by a stress test reported as 93% (126 of 136 patients) at a mean of 30 months follow-up. Subjectively, the short- and long-term cure rates were 95.3% and 90.4%, respectively.[21] The introduction of the minimally invasive midurethral slings using a monofilament polypropylene material for SUI in the mid-1990s has led to this material being largely abandoned.

As it was used for a number of years, surgeons may still encounter postoperative complications, namely erosions with this particular product.

### Polypropylene mesh

Polypropylene was created in 1955 by F. J. Natta. It is a thermoplastic polymer which has a variety of applications including food packaging and car components. When used in a mesh, this material is composed of loosely woven strands of synthetic material. The suggestion here is that a pore size greater than 80 μm supposedly allows the passage of macrophages which may result in improved host tissue ingrowth when compared with its smoother, more tightly woven equivalents and thus reduces infection.

The tension-free vaginal tape^®^ (TVT; Ethicon, New Brunswick, NJ) is a polypropylene monofilament mesh, which is a very commonly used material today. Long-term follow-up (seven-year data) has been reported with good results.[23] Cure rates were 84.6%, although satisfaction rates were lower at 69.3%. The cure rates were lower in patients with high-grade SUI (50% in Grade III, 82.8% in Grade II and 90.7% in Grade I; *P* < 0.001). This was supported by further long-term studies[24,25] which reported success rates of 81.3% and 79.2% respectively at seven and five years. None of these studies reported any cases of tape erosion or infection.

More recently, the transobturator technique (TOT) and the single-incision mini-sling have been reported in attempts to further reduce the risks of sling placement. Polypropylene slings have also been used via this transobturator route. A recent meta-analysis of all published studies between 2008 and 2009 comparing TVTs and TOTs showed that the short-term objective cure rate was slightly better in the TVT group [odds ratio (OR) 0.62; 95% confidence interval (CI) 0.37-1.00; *P* = 0.05].[26]

### Polytetrafluoroethylene

Polytetrafluoroethylene (PTFE) was discovered by Roy Plunkett at DuPont in 1938 and introduced as a Commercial product (Teflon) in 1946. A process where the PTFE is thermomechanically expanded turns it into a microporous material. The end result is a soft and pliable multifilament mesh known as Gore-Tex, which is said to cause fewer adhesions and a less obvious inflammatory response.[27] Gore-Tex has been classified as a Type II biomaterial with a pore size smaller than 10 μm.

Early success rates with a Gore-Tex patch sling were encouraging, with success rates between 83% and 89%.[28,29] However, there were problems with erosion and rejection rates reportedly as high as 37.5% with this material, which has diminished its use.[30]

### Peri-urethral injectables

The increasing demand for minimally invasive options for SUI has resulted in the development of agents and techniques that improve these conditions substantially towards social continence, however, the existing cure rates are suboptimal. As always, correct patient selection is vital. The model patient is one who has good anatomical support, a compliant, stable bladder, and a malfunctioning urethra, evidenced by a low leak-point pressure.[31]

Since 1938, surgeons have been attempting to treat urinary incontinence by injecting the urethra. The first substance used was sodium morrhuate and subsequent to this a number of materials have been trialed. They include PTFE in the form of Teflon; glutaraldehyde cross-linked (GAX) collagen (Contigen); silicone (polydimethylsiloxane, Macroplastique) and autologous fat. The ideal properties for these materials should be biocompatibility, minimal immune reaction, no separation of agent subcomponents, minimal host response, reproducible characteristics, minimal fibrotic ingrowth, little extracapsular inflammatory response and minimal resorption.[32]

Two of the most commonly used agents are as follows:

*Bovine GAX-Collagen (Contigen, C.R. Bard Inc., Murry Hill, NJ)* Over the last decade, GAX-collagen has become the most widely used injectable in stress incontinence. The material is purified from bovine dermis into an acellular derivative, before being treated with enzymes to remove telopeptides thus decreasing antigenicity. It is then cross-linked with glutaraldehyde to withstand breakdown from host collagenases.

After injection, the implant is neovascularized which encourages the active invasion of host fibroblasts. New, endogenous collagen is then manufactured within the implant promoting physiologic stability.[33]

There are many studies evaluating this material’s effectiveness, unfortunately, the lack of standardization in ‘cure rates’ throughout, means it is difficult to draw strong conclusions. One long-term follow-up study of 50 months reported cure and improvement rates of 30% and 40% respectively.[34] However, 33% patients required more than one injection to achieve success.

A recent multicentred, randomized controlled trial (RCT) comparing the use of contigen and macroplastique on 122 patients found that at 12 months the macroplastique group had a superior cure rate of 36.9% compared to 24.8% in the contigen group (*P* <0.05).[35] Further standardized studies are required to draw firm conclusions

## SILICONE – MACROPLASTIQUE

This material is a soft tissue bulking agent and is comprised of soft, flexible, highly-textured irregularly shaped implants of heat-vulcanized polydimethylsiloxane (a solid silicone elastomer) suspended in a bio-excretable carrier gel. The silicone element is well-tolerated by the cellular immune system and is non-genotoxic, non-carcinogenic and non-teratogenic. Because of its irregular shape and textured surface, agglomeration and host collagen deposition are enhanced and encouraged. Injected particles are organized within six to eight weeks in firm nodules with infiltrated collagen and surrounded by a fibrous sheath.[36] Sixty-month follow-up has shown objective success rates to be 80% in women with urodynamically proven intrinsic sphincter deficiency.[37]

A meta-analysis of all the published studies investigating silicone injection treatments for SUI in adult women found low methodological quality of included studies. The authors speculated that results should be interpreted with caution and no firm conclusions about the efficacy of silicone was possible.[38]

### Zuidex

This agent is a cross-linked substance consisting of dextranomer microspheres which have been cross-linked with hyaluronic acid (HA). This gel has a highly elastic consistency with a high viscosity level. It is completely biodegradable and not immunogenic. The HA is resorbed within two weeks after injection, whereas the dextranomer microspheres which act as the bulking agent, remain in the injection site for four years. The gel is injected with an ‘Implacer’ at the mid-urethra and does not require even standard cystoscopic equipment. It has been investigated in two European studies, both involving surgery-naive patients in whom intrinsic sphincter deficiency or hypermobility was not determined.[39,40] In the first study (n = 42), significant improvements in median provocation test urine leakage and number of incontinence episodes over 24 h were observed at 12 months (both *P* < 0.0001 versus baseline), with 24% reporting no leakage as assessed by provocation test. Improvements were sustained to 24 months in a follow-up population (n = 20). In the second study (n = 142), a >50% reduction in provocation test urine leakage versus baseline was observed in 73% of patients at six months, with 33% dry (< 1 g leakage). In addition the median number of incontinence episodes/24 h decreased from 3.0 at baseline to 0.8 at six months (*P* < 0.0001). These improvements were sustained out to 12 months.

### Intradetrusor injections

Botulinum Toxin (BTX) A is proving to be one of the most significant recent developments in the treatment of the overactive bladder.[41] BTX is produced by the spore-forming bacterium *Clostridium botulinum*, which is an obligate anaerobe. There are seven distinct types of toxin (labeled A-G) which have been isolated, however, only types A and B have been widely used in the clinical setting.[42] They are all proteins which have a similar molecular structure and weight (140-170 kDa). The toxins are initially synthesized as single-chain polypeptides, but then via a two-step process they are split into a light and heavy chain held together by a disulphide bond. The light chain is a toxic zinc protease unit and the heavy chain is a hemaglutinin. The serotypes vary in the extent that they are cleaved and activated.[43] The toxin binds tightly to the intramuscular nerve terminals and blocks the presynaptic acetylcholine release leading to a flaccid paralysis. Although this is true it seems likely that the toxin also affects the vesicular release of neurotransmitters involved in the afferent arm of reflex bladder contractions.[44]

Injection of this neurotoxin into the detrusor muscle leads to improved bladder capacity and compliance and reduction of the urgency associated with neurogenic bladder. Patients generally report symptomatic improvement as early as one to two weeks after injection, and the effects last between 6-11 months.[45]

## COMPLICATIONS

### Slings

#### Sling erosion

Sling erosions have been reported through the urethra, bladder and less commonly through the vagina. Urethral erosion (UE) is an uncommon but potentially severe complication after suburethral synthetic sling insertion. Early slings used a bone anchor system with a polypropylene mesh (Vesica; Boston Scientific, Natick, MA). This was then followed in 1996 by a bone anchor sling using a polyester weave impregnated with bovine collagen (ProteGen; Microvasive, Natick, MA). Both were removed from the market by 1999 due to high rates of mesh erosion, infection, pain, and urethrovaginal fistula formation.[46]

A large meta-analysis of all published sling-related complications[47] found 19 studies describing erosion/extrusion in 2197 patients. The overall incidence was 6%; this was broken down further to give incidence rates of 2.6-4.8% in TVTs, 7.7% in polyester mesh and silicone materials and a rate of 1.3–20% for TOTs. A multi-institutional review in 2004 of 241 patients found the erosion rate for their TVTs to be 0.4%.[48] Problems with many of these studies relate to the difficulty in discriminating extrusions from true mesh erosions, as well as the anatomical site of erosion (i.e. vaginal or urethral).

Risk factors identified with urethral erosion have been identified are excessive tensioning of the sling, a peri-operative urethral perforation and one or more postoperative urethral dilatations.[49]

The correct management for erosions is still uncertain. Initially, conservative treatment should be trialed. However, if this leads to further infections or pain surgical intervention should be carried out. This may be in the form of removing the sling completely or excising the infected portion via an open technique. Transperitoneal laparoscopic removal of eroded slings has also been reported.[50,51] This minimally invasive removal allows for better exposure of the whole sling as well as allowing for a quicker recovery.[52] More recently, laser removal of eroded tapes has also been reported.[53,54]

## DETRUSOR INSTABILITY

It has been shown many times in the literature that women who have mixed incontinence and are treated with sling surgery have a chance of resolution of their bladder urgency. A recent study of 305 women with mixed symptoms, reported that 31.5% had resolution of the urgency,[55] with increased improvements in patients undergoing a TOT procedure (53% of patients showing a positive response).

The rate of *de novo* detrusor instability has been reported between 5.9–25%.[56] A comparison of improvement of urgency as well as rates of *de novo* urgency in 276 women undergoing different sling procedures, found that women who undergo TOT procedures have significantly lower rates of *de novo* urge incontinence than those who undergo midurethral sling procedures. However, this study found that rates of resolution of prior detrusor overactivity did not differ between groups.[57]

The cause of *de novo* urgency has been suggested to result from the combination of mild bladder outflow obstruction and urethral irritation caused by the sling. Abouassaly *et al*.’s multicentered trial (2004) found *de novo* rates in women undergoing TVT procedures to be as high as 15%.

## URINARY RETENTION

Obstructive complications after incontinence surgery are well documented. Although the TVT tape is inserted without tension, urinary retention is well documented after this surgery. Of 241 undergoing TVTs, Abouassaly *et al*. quoted rates of 19.7%. A large randomized control trial investigating preoperative urodynamic findings in order to predict postoperative voiding dysfunction after pubovaginal sling (the exact type of sling was unfortunately not specified) or Burch colposuspension, found that of the 655 women randomized, 57 developed voiding dysfunction; eight in the Burch colposuspension and 49 in the pubovaginal sling groups.[58]

As with most of the incontinence studies, a lack of standardization of voiding dysfunction and disparity within the women investigated have led to a wide range of reported retention rates. A recent meta-analysis of published papers from 2008 to 2009 regarding women undergoing different sling procedures, fount that there was an increased risk of retention in the TVT group when compared to TOT (Odds ratio 1.6, *P* < 0.06).[26]

The rates in TOT slings have been reported as lower in a variety of papers, but again the rates vary from 0–15.6%.[59,60] The treatment options of an indwelling or intermittent self-catheter should be trialed as the retention often settles spontaneously. If there is no improvement after a month, early simple sling lysis should be considered.[61]

## INFECTIONS

Nonspecific pelvic pain, persistent vaginal discharge or bleeding, dyspareunia, and urinary or fecal incontinence are the most common manifestations of vaginal mesh-related infection. Clinical examination may reveal induration of the vaginal incision, vaginal granulation tissue, draining sinus tracts, and prosthesis erosion or rejection. Various pathogens have been implicated, including Gram-positive and Gram-negative aerobic and anaerobic bacteria. The management of infections in women who undergoing incontinence surgery is combined surgical and medical treatment.

There are numerous postoperative complications published. These range from urinary tract infections,[62] abscess formation in an assortment of anatomical spaces (thigh to ischiorectal fossa), as well as fistulas, necrotizing fasciitis, osteitis pubis and systemic sepsis, and urinary tract infection.[56,63,64] Infectious complications may be delayed by several months or years after the sling procedure.

## PAIN

As the TOT trocar passes close to the obturator nerve within the canal, injury and subsequent leg pain is a real possibility. Thigh pain has been documented in 5% of 117 women undergoing this procedure.[65] This is also supported by a Finnish RCT, which reported postoperative pain rates of 16% for TOT versus 1.5% for TVT postoperatively postoperatively.[60]

Dyspareunia is also a recognized complication. Rates up to 7.3% have been recorded in TVT patients and 1.3% in TOT patients.[66,67] This is particularly important when more than half the women undergoing these procedures are sexually active.[68]

In regard to SIS slings, postoperative inflammation resulting in pain and induration at the abdominal incision site was reported in six out of 10 women undergoing an SIS pubovaginal sling.[69] Symptoms presented 10 to 39 days postoperatively. All were managed with medical treatment apart from one patient requiring incision and drainage of an abscess.

## BLEEDING

Hemorrhage can arise in the thigh, vulva, retropubic spaces, abdomen and pelvis. The incidence of recognized bleeding following TVT is 0.7–8 %.[70,71] The mean distance from the major vessels and the trocar at TVT has been measured as 3.2–4.9 cm.[72] One patient has been reported as having died secondary to an arterial bleed following surgery.[56] Treatment is generally conservative, but if there is evidence of an expanding hematoma causing pain or infection, draining may be necessary.

## BOTULINUM TOXIN

In general the urological applications of this toxin are small (200-300 u) and it is understood that a lethal dose in humans would be 3000 u, therefore it is thought to be unlikely that any serious systemic muscle paralysis would occur. In addition, BTX does not cross the blood-brain barrier so direct central nervous effects are not seen. However, there have been reports of generalized muscle weakness. Dykstra and Sidi[73] have reported three cases of upper arm weakness in SCI patients who received 140-240 u Botulinim Toxin Type A into their urethral sphincter. The weakness resolved after two to three weeks. Similar findings were found in five out of 61 patients with DSD who received either 300 u Botulinim Toxin Type A or 1000 u of Dysport, and again the symptoms resolved after one month.[74] This particular study also reported that four patients complained of visual disturbances, but these disappeared after oral anticholinergics were stopped. Two cases of distal muscle weakness after bladder injections have been presented in the literature,[75] however, in these two cases the weakness lasted three months. Although the reported weaknesses were not life-threatening, this type of side-effect could be particularly debilitating for patients who already have some form of disability.

The possibility of incomplete bladder emptying cannot be understated and the resultant need to perform clean intermittent self catheterisation must be agreed by the patient. Data from one of the largest idiopathic detrusor overactivity studies[76] found a rate of 4%. However, they used the smaller value of 100 u in their patients, with only 88% of their patients experiencing a significant improvement in their urinary symptoms. CISC rates have been reported as high as 45%.[77] A recent RCT has described rates of 26% of patients found to have post-void residual volumes of 200 cc or greater and one subject required intermittent catheterization.[78] There are large discrepancies between different studies and rates of post-void residuals, but this can usually be explained by the use of different doses, preparations or injection sites.

A final consideration is that BTX is not a cure; patients will have to undergo repeat injections with the possibility of developing resistance to the drug. Patients normally have relief of symptoms for 9 to 12 months.[79–81] At present there is little long-term data examining the effect of repeat injections. It has been hypothesized that this progressive re-innervation may eventually enhance the pathological innervation that these patients experience, thereby exacerbating their symptoms.[82]

## CONCLUSIONS

The central aim in incontinence surgery is to restore anatomy and function whilst doing the least harm to the patient. Modern biomaterials are allowing us to explore further surgical options be it grafts, peri-urethral injectables and even intra-detrusor treatments. Contemnor surgical procedures need additional, longer follow-up so that long-term complications as well as overall effectiveness can be measured.
